# Three-Toed Sloth as Putative Reservoir of *Coxiella burnetii*, Cayenne, French Guiana

**DOI:** 10.3201/eid2010.140694

**Published:** 2014-10

**Authors:** Bernard Davoust, Jean-Lou Marié, Vincent Pommier de Santi, Jean-Michel Berenger, Sophie Edouard, Didier Raoult

**Affiliations:** Aix-Marseille Université, Marseille, France (B. Davoust, J.-L. Marié, J.-M. Berenger, S. Edouard, D. Raoult);; Groupe de Travail en Épidémiologie Animale du Service de Santé des Armées, Toulon, France (J.-L. Marié);; Direction Interarmées du Service de Santé en Guyane, Cayenne, France (V. Pommier de Santi);; Centre d’Épidémiologie et de Santé Publique des Armées, Marseille (V. Pommier de Santi).

**Keywords:** Amblyomma geayi, Bradypus tridactylus, Coxiella burnetii, French Guiana, Q fever, sloth, 3-toed sloth, tick, bacteria, outbreak, zoonosis

**To the Editor:** Q fever is an emerging zoonosis and a major public health concern in French Guiana, a French overseas region located on the northeastern coast of South America (*1*,*2*). Most cases occur in the city of Cayenne (*3*), specifically in the suburbs, where houses are near wooded hills (*4*). Genotyping performed by using multispacer sequence typing showed that MST17, a unique genotype of *C. burnetii*, circulates in Cayenne and is responsible for epidemics of Q fever (*5*). *C. burnetii* transmission peaks during the rainy season, and the incidence of Q fever usually increases 1–3 months later (*6*). The animal reservoir of *C. burnetii* in French Guiana is unknown; previous studies have excluded domestic ruminants, which are known to be *C. burnetii* reservoirs elsewhere in the world (*6*). Four serologic surveys showed few *C. burnetii*–positive opossums, dogs, rodents (*Proechimys* spp*.*), bovines, or birds in French Guiana (*7*). In 2013, using real-time PCR (qPCR) analysis of vaginal swab samples, we showed that 6/158 (3.8%) dogs from Cayenne and 0/206 bats from the coastal area of French Guiana were positive for *C. burnetii* (cycle threshold [C_t_]<35). One of the positive samples was identified as genotype MST17 (*5*). A case–control study among humans identified several risk factors for Q fever, including living near a forest and the presence of wild animals near the house (*6*).

During January–April 2013, a Q fever outbreak occurred in Tiger Camp, a military residential area located at the top of a wooded hill in Cayenne. Vaginal swab samples were collected from animals living in the area (13 goats, 8 sheep, 7 bats, 34 birds, 2 opossums, 4 iguanas, and 17 geckos); all samples were negative for *C. burnetii* by qPCR. In addition, serologic tests for *C. burnetii* were negative for samples from all 37 small ruminants maintained near the outbreak area.

In January 2014, a dead (accidental death) female 3-toed sloth (*Bradypus tridactylus*) (Figure, panel A) was found on the road near the residence of a Q fever patient. We retrieved the sloth and collected feces, spleen, liver, kidney, lung, and uterus samples and a vaginal swab sample. A total of 16 ticks were removed from the sloth and stored in 70% alcohol. 

**Figure:**
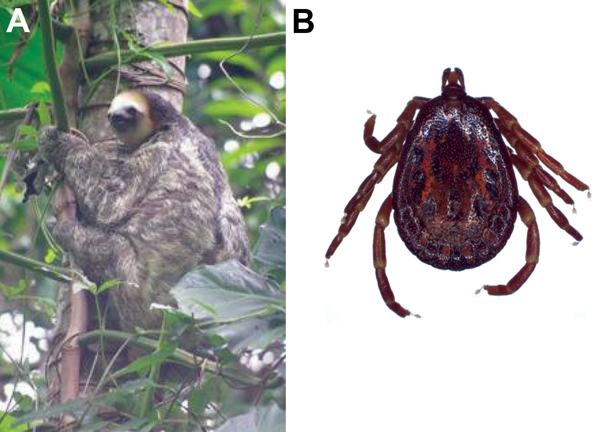
Putative reservoir of *Coxiella burnetii* in Cayenne, French Guiana A) A 3-toed sloth (*Bradypus tridactylus*) in its natural habitat in Tiger Camp, a military residential area located at the top of a wooded hill in Cayenne, French Guiana (photograph by S. Fernandes). B) A male tick (*Amblyomma geayi*) found on the 3-toed sloth in this study (photograph by J.M. Berenger).

DNA was extracted from the feces, organs, and ticks by using the BioRobot EZ1 Workstation (QIAGEN, Courtaboeuf, France). qPCR targeting the repeated insertion sequence IS*1111* was performed by using a CFX96 Touch Real-Time PCR Detection System (Bio-Rad, Marne la Coquette, France) as described (*8*). We confirmed all positive results by performing a second qPCR targeting the IS*30a* repeated sequence. DNA samples with C_t_ values <35 in both assays were considered positive for *C. burnetii*. A standard calibration curve quantifying the target IS*1111* was generated by using 10-fold serial dilutions of *C. burnetii* Nine Mile strain. The number of IS*1111* intergenic sequences found in the genome of strain *C. burnetii* MST17 was identical to that for the Nine Mile strain (F. D’Amato, unpub. data); thus, the qPCR that we used was valid for quantifying the number of *C. burnetii* MST17 IS1111 copies/mL in samples we collected (*5*).

qPCR analysis showed that the feces were highly positive for *C. burnetii*; the sample had a low C_t_ value of 23, corresponding to 7 log_10_ DNA copies/mL (*9*). The spleen was also positive for *C. burnetii*; the C_t_ value was 34, corresponding to 3.6 log_10_ DNA copies/mL. Results for the other samples were negative. 

Using morphologic criteria, we identified all 16 ticks collected from the sloth as *Amblyomma geayi* (Figure, panel B). We performed *C. burnetii*–specific qPCR on the ticks; 14 (88%) were positive. 

We genotyped *C. burnetii*–positive DNA from the feces and from 6 of the 16 ticks by using multispacer sequence typing as described (*5*). All samples were identified as MST17, the unique genotype circulating in Cayenne (*5*).

After obtaining the laboratory results, we confirmed that a local group in charge of the collection and treatment of injured animals usually released rehabilitated 3-toed sloths into Tiger Camp. Residents of Tiger Camp regularly observed and came into contact with the sloths, and ticks were frequently observed on the fur of the animals. Furthermore, 3 Q fever patients from Cayenne reported contact with sloths.

Feces from the sloth in this study were highly infectious for *C. burnetii*. Because sloths live in tall trees and can shed this bacterium in their feces, human contamination might occur through inhalation of infectious aerosols from feces. The high prevalence of *C. burnetii* infection in ticks also suggests possible transmission through tick bites or from aerosols of tick feces that have been deposited on the skin of animal hosts; such feces can be extremely rich in bacteria and highly infectious (*10*).

In this 2013 outbreak of Q fever, epidemiologic studies led to the identification of 3-toed sloths as a putative source of *C. burnetii* infection. Further investigations are needed to confirm the role of sloths as a reservoir for *C. burnetii* in French Guiana and to implement efficient measures to prevent transmission to humans.
